# The assessment of reservoir potential of Permian to Eocene reservoirs of Minwal-Joyamair fields, upper Indus basin, Pakistan

**DOI:** 10.1016/j.heliyon.2023.e16517

**Published:** 2023-05-22

**Authors:** Muhammad Ali Umair Latif, Muhsan Ehsan, Muhammad Ali, Abid Ali, Armel Zacharie Ekoa Bessa, Mohamed Abioui

**Affiliations:** a Department of Earth and Environmental Sciences, Bahria University, Islamabad, Pakistan; b Institute of Geophysics & Geomatics, China University of Geosciences, Wuhan 430074, PR China; c Institute of Geology, University of the Punjab, Lahore, Pakistan; d Institute of Earth Sciences (ISTE), University of Lausanne, Lausanne, Switzerland; e Geosciences, Environment and Geomatics Laboratory (GEG), Department of Earth Sciences, Faculty of Sciences, Ibnou Zohr University, Agadir, Morocco; f MARE-Marine and Environmental Sciences Centre - Sedimentary Geology Group, Department of Earth Sciences, Faculty of Sciences and Technology, University of Coimbra, Coimbra 300-456, Portugal

**Keywords:** Carbonate reservoirs, Data conditioning, Seismic interpretation, Petrophysical analysis

## Abstract

Upper Indus Basin has been a valuable asset as the complexity of structure and hydrocarbon production is the leading producer of oil and gas in history and still to date. Potwar sub-basin has significance in the light of oil production from carbonate reservoirs or Permian to Eocene age reservoirs. Minwal-Joyamair field is very significant and has unique hydrocarbon production history with complexity in structure style and stratigraphy. The complexity is present for carbonate reservoirs of the study area due to heterogeneity of lithological and facies variation. In this research, the emphasis is on integrated advanced seismic and well data for Eocene (Chorgali, Sakesar), Paleocene (Lockhart), and Permian age (Tobra) formations reservoirs. This research's primary focus is to analyze field potential and reservoir characterization by conventional seismic interpretation and petrophysical analysis. Minwal-Joyamair field is a combination of thrust and back thrust, forming a triangle zone in the subsurface. The petrophysical analysis results suggested favorable hydrocarbon saturation in Tobra (74%) and Lockhart (25%) reservoirs in addition to the lower volume of shale (28% and 10%, receptively) and higher effective values (6% and 3%, respectively). The main objective of the study is the re-assessment of a hydrocarbon producing field and describe the future prospectively of the field. The analysis also includes the difference in hydrocarbon production from two different type of reservoir (carbonate & clastic). The findings of this research will be useful for any other similar basins around the world.

## 1 Introduction

**Fig. 1:**
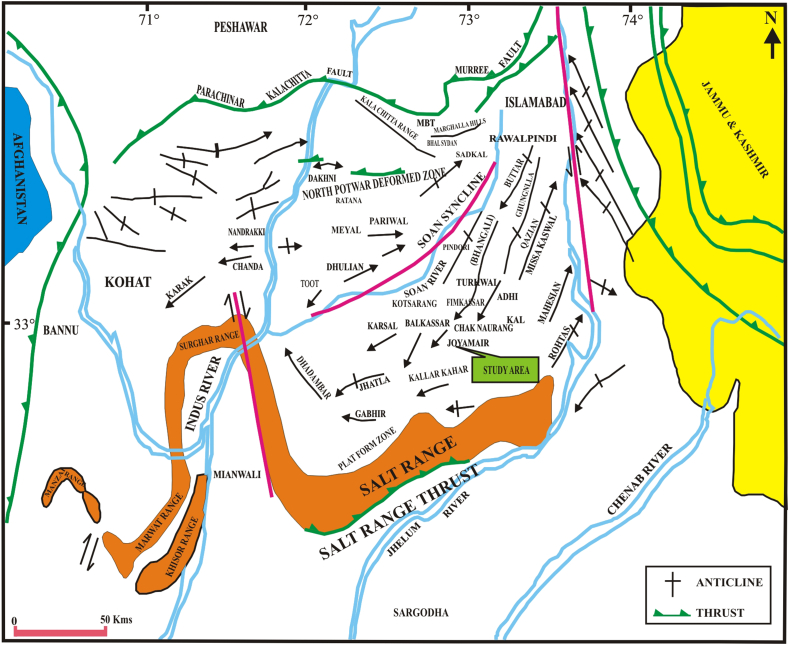
The generalized structural and location map shows the position of Minwal-Joyamair with adjoining fields [1].

Potwar Sub-Basin lies in the Upper Indus Basin (UIB), which has a very complex tectonic and structural setting. This region is the main hydrocarbon producer, but the structural, digenetic, and lithological complexity of the area makes it more difficult for oil and gas exploration by only conventional methods. For this reason, advanced exploration and production techniques are to be carried out for better productivity and understanding of reservoir potential [1]. Researchers suggested advanced methods for further hydrocarbon potential evaluation, but no detailed work has been done for any conclusion. Apart from all the above difficulties, Eastern Potwar's fields are the leading producers of hydrocarbon production. Minwal-Joyamair fields (Study Area) as one of the old fields present in Eastern Potwar which is adjacent to many producing fields such as Balkassar, Adhi, Chak Naurang, Pindori, and Turkwal, etc. as shown in Fig. 1.

The structures of the Joyamair area are very complex due to folding and faulting, resulted due to stresses caused by the collision of Indian and Eurasian plates [2]. Seismic interpretation of the available 2D seismic, gravity and magnetic data reveals that tectonically, the study area has undergone severe deformation, which consists of thrusts and back thrusts. Minwal-Joyamair consists of one structure, which appears to be an open anticline on the surface. It consists of thrust and back thrust, which forms a triangle zone in the subsurface [[1], [2], [3], [4]]. Three phases of fractures are recorded in the area. The latest phase of fractures is open. These open fractures are the result of secondary porosity in these carbonates reservoirs in the Minwal-Joyamair field [5,6].

The Eocene, Paleocene, Permian, and Cambrian age formations produce oil in the Eastern Potwar sub-basin [7]. The primary porosity subordinates to clastic and secondary porosity due to fractured carbonates in these reservoirs. Chorgali and Sakesar formations of carbonates are the major hydrocarbon producers in the Minwal-Joyamair area. The Sakesar Limestone is partly dolomitized and contains chert nodules. The Chorgali Formation is partly dolomitized with thin beds of limestone [1,8,9]. Lockhart Limestone may be considered a reservoir with good, fractured porosity [10]. Tobra Formation has poor to moderate reservoir quality based on well cutting study in the Potwar sub-basin [11].

Worldwide, well logs and seismic data are utilized to understand reservoir properties and subsurface structural traps [12,13]. Cross-plot analyses and numerical equations are very helpful for a better understanding of formation evaluation based on well logs data set [[14], [15], [16]]. The seismic imaging method provides an invaluable tool for petroleum exploration and optimizing the efficient production of developed petroleum fields [17]. To evaluate reservoir potential for the integrated study well logs, interval velocities, seismic data, petrophysical modeling, and seismic characteristics are valuable tools [18,19].

Pakistan is currently facing a severe energy crisis due to the increase in demand of hydrocarbon to generate power. Present oil and gas resources are depleting day by day which are putting pressure on exploration and production companies to explore the new resources and exploit the present resources at maximum. Therefore, a re-evaluation studies must be performed to meet the demands of the country. Several studies are conducted across the globe to assess these resources using 2D and 3D seismic data, and well log data [[20], [21], [22]]. Advanced exploration techniques are carried out to increase oil and gas production in the field through the integration of seismic and well data to enhance the precision of the interpretation process [23]. Seismic and well logs data conditioning was performed (5, 6, & 7). Mistie's calculation was performed due to different seismic vintages. The well-to-seismic tie was performed to better seismic interpretation of 2D seismic data, which helps to understand the subsurface structural geometry. In this research integrated approach seismic interpretation, and petrophysical analysis was performed to better understand of reservoir potential of Permian to Eocene reservoirs of Minwal-Joyamair fields, Upper Indus Basin, Pakistan. This research will emphasize any possible outcomes to enhance hydrocarbon productivity for these types of complex oil and gas fields.

## 2 Geological settings and stratigraphy

UIB is in the North of Pakistan, which is divided from the Lower Indus Basin by Sargodha High. In the North and East is the “Main Boundary Thrust (MBT)” [24]. The region expands on an area of 120 km from the MBT (North) and Jhelum River (South). The UIB is further subdivided into Potwar (East) and Kohat (West) Sub-Basin, which the Indus River separates. Both sub-basins depict tectonically deform and facies variations (Fig. 1). Potwar Sub-Basin preserves the sedimentary rocks of the Precambrian to Quaternary age, which are entirely exposed in the Salt Range [5,25].

Adhi and Chak Naurang oil fields are located in the surrounding Joyamair, which are producing hydrocarbons from Khewra and Tobra formations. Thus, Minwal-Joyamair field re-evaluation especially for Tobra can be very much productive. Time and depth maps were generated at the top of the Tobra Formation, which is a good producer of hydrocarbons in the surrounding fields (Chak Naurang and Adhi). The well-established structural closure, proven reservoir parameters, and earlier testing results confirm the hydrocarbon potential of the reservoirs [1].

**Fig. 2:**
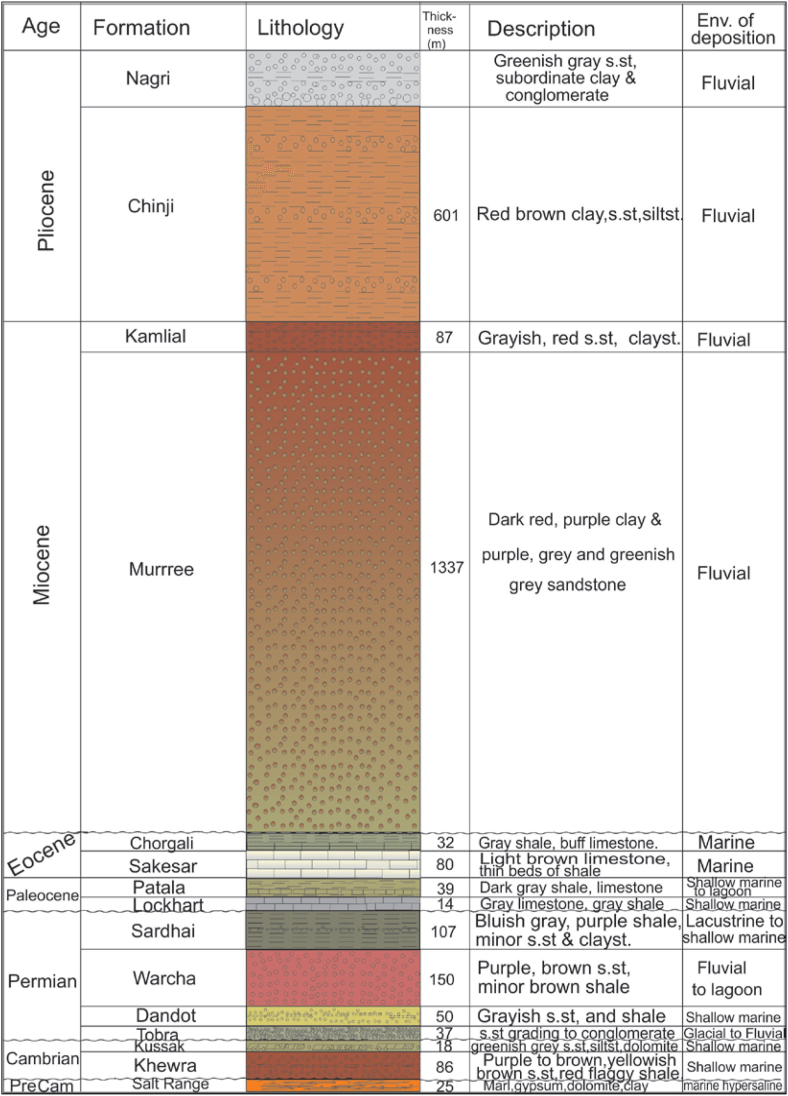
Generalized stratigraphic column for Eastern Potwar [5].

The deposition of the Kohat-Potwar geologic province from the Late Proterozoic to the Holocene is completed. The basement of the basin consists of Late Proterozoic metamorphic rocks. Where there is the deposition of shales, sandstones, and evaporate of Cambrian Salt Range Formation and Proterozoic age formations (Fig. 2). There is a deposition of carbonates in the uppermost part of the Salt Range and the lower part evaporates. Oil shows in these evaporate to indicate source rock potential [24]. A brief description of the drilled stratigraphic column is given in Fig. 2 [7].

## 3 Data set and methodology

**Fig. 3:**
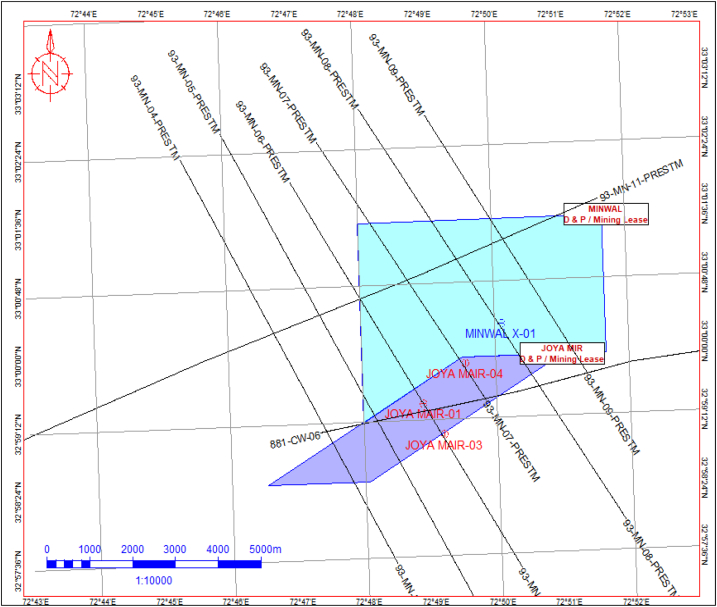
Seismic base map along with wells location.

The available seismic data consist of eight (08) 2D lines of PSTM version and vintage of 1993 and 1981 and well data were available of Minwal X-1, Joyamair-1, Joyamair-3, and Joyamair-4. The details analysis was performed only on Joyamair-4 due to the availability of desired logs of Permian to Eocene reservoirs. The quality of available seismic lines was moderate to low fold data where structural interpretation was possible, but advanced/quantitative interpretation has some limitations. These 2D seismic lines were used to interpret prospective horizons (formations) and faults (Fig. 3).

### 3.1 Seismic data interpretation

On the seismic lines, prominent reflectors were identified based on the continuity of reflectors [4]. Reflectors correspond with horizons mark the boundary between rocks of different lithology. However, it may be simply a seismic marker horizon that occurs close to that boundary. This problem can be resolved by the correlation of seismic and borehole data and synthetic seismograms. Seismic data have become the key tool used to explore hydrocarbon [26].

**Fig. 4:**
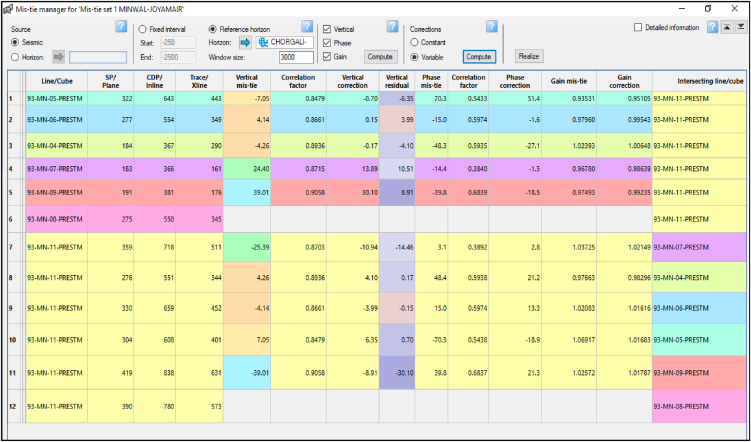
Mistie calculation for available 2D seismic lines.

**Fig. 5:**
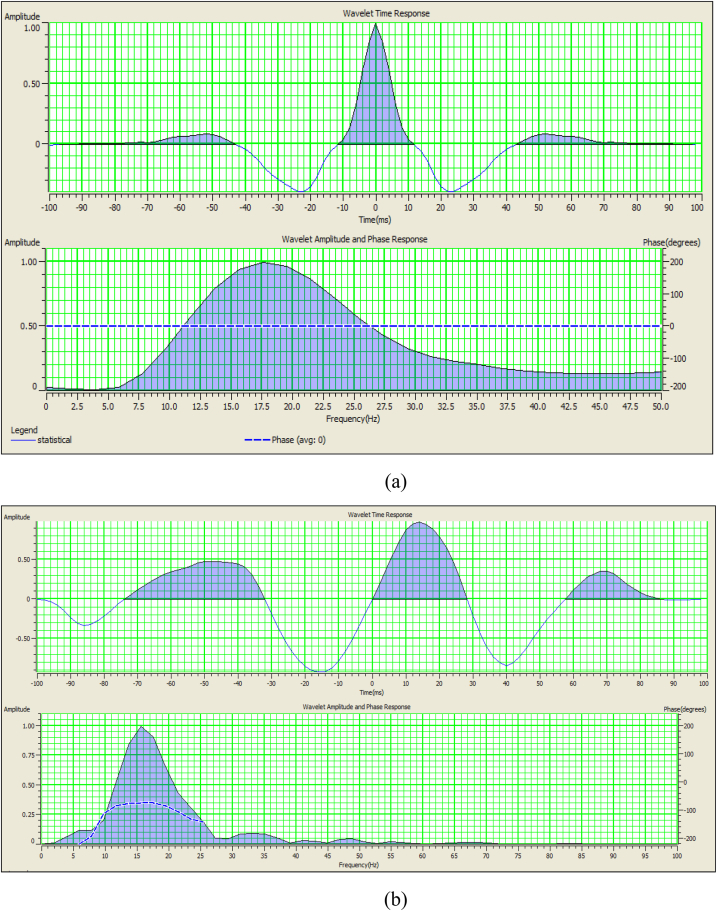
(a) Statistical Ricker wavelet used for synthetic generation. (b) Wavelet extracted using well log data.

**Fig. 6:**
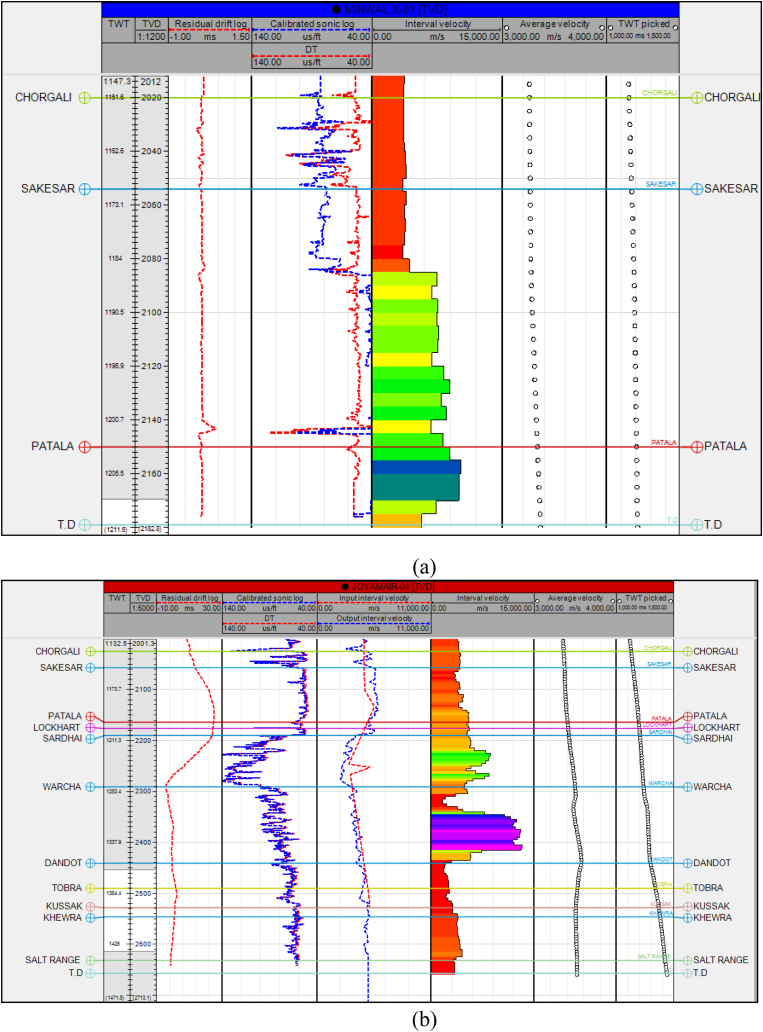
(a) Minwal X-1 sonic calibration. (b) Joyamair-4 sonic calibration.

As mentioned above, the analysis of seismic records implies processes, namely, identification, picking, and correlation determination of structure from the conversion of reflection time into depth. The following steps were followed to perform the seismic interpretation in this study which includes identification of reflectors (horizons), mistie calculation (Fig. 4), horizon continuity, defining plausible fault geometry, well to seismic tying, wavelet extraction (Fig. 5), and calibration of seismic with the sonic log (Fig. 6(a and b)). Ricker [27] selected a point where the signal is flat or has zero value of curvature (Fig. 5a). This flat point occurs when the time division equals *1/(2.3F),* where *F* is the predominant frequency [28]. The choice is dependent on the available frequency spectrum of data. Find acoustic impedance data from a well and convert the logs to synthetic curves as a function of time (Fig. 5b).

### 3.2 Velocity modeling

**Fig. 7:**
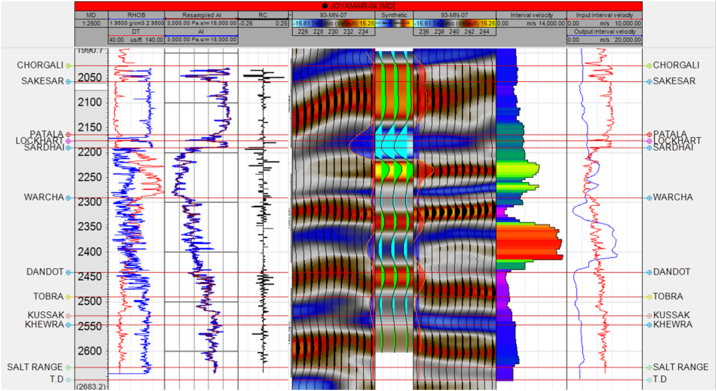
Synthetic generation for Joyamair-04 well.

Some forms of velocity functions and linear acceleration functions of the form V_0_ + k × Z (where V_0_ = reference velocity, k is the velocity gradient, and Z is depth) are significant for seismic modeling [28]. A good quality seismic image is not the only tool required for an exploration or field development interpretation. Enhancing exploration also involves combining good well ties and accurate depth conversion [29]. Depth conversion can characterize into two main parts, time-to-depth conversion by the direct method and velocity modeling [30]. Both approaches, when used successfully, can reliably tie existing wells together and precisely estimate depth [28]. Synthetic generation for the Joyamair-04 well is shown in Fig. 7.

### 3.3 Petrophysics

Carbonate reservoirs have a different approach for the calculation of water saturation, as in Archie's equation, as compared to clastic. Clastic reservoir saturation calculations tend to be predictable whereas carbonate has different approaches. Archie's saturation equation uses two exponents, cementation factor (m) and saturation exponent (n), which for clastic rocks can be equal to 2. On the other hand, this estimation of exponent value can be complex for carbonate reservoirs [31]. Well-log responses were used to help identify the different lithologies and lithofacies [32].

In this study, V_sh_ has been calculated by linear equation method whereas V_sh_ estimated from the cross-plot method is validated with Vsh calculated from gamma ray log (CGR). The volume of shale (V_sh_) is calculated by an equation (1), (2) by Poupon and Gaymard (1970) [33] which introduced linear equations.

(1) $$I_{GR}\frac{{GR}_{\log}-{GR}_{\min}}{{GR}_{\max}-{GR}_{\min}}$$

Where, I_GR_ = Gamma Ray index, GR_log_ = Gamma Ray log values, GR_max_ = Gamma Ray maximum values, and GR_min_ = Gamma Ray minimum values

(2) $$I_{GR}=V_{sh}$$

Chorgali and Sakesar formations, as the main reservoir in the Eastern Potwar, have very low to moderate parts of shale present in them. Thus, and the limitation of available well-log data and it is processing, uncertainty changes can be present.

Porosity is a measure of the available pore spaces between sediments regarding the matrix/cement of rock. In carbonates, porosity can be from 1% to 30%, where dolomite has 10%, and limestone is around 12% [34]. In other words, Porosity can be defined as the fraction of spaces not occupied by sediments [35].

The average porosity is calculated by density and neutron logs. The electron density of the rock unit is measured by the density log. The basic purpose of this log is to spot gas zone, limit the hydrocarbon density, and categorize evaporate minerals, shaly sand reservoirs evaluation, and lithology composite [36]. Porosity derivation using density logs incorporates by equation (3).

(3) $${Ф}_{D}=\frac{\rho_{ma-\rho_{b}}}{\rho_{ma-\rho_{f}}}$$

Where, ${Ф}_{D}$ = density derived porosity, $\rho_{ma}$ = matrix density, $\rho_{f}$ = fluid density (1.0 Fresh Water 0.7 gas and 1.1 salt mud) and $\rho_{b}$ = bulk density values from log

Effective porosity ${Ф}_{E}$ is therefore described by equation (4).

(4) $$Effectiveporosity=Average\;porosity\;\left(1-V_{sh}\right)$$

Saturation is the spaces occupied by water (water saturation; S_w_) or hydrocarbon (Saturation of hydrocarbon; S_h_). It can be estimated by different methods [37] and given as a percentage. In this study, a saturation of water (S_w_) is calculated by Archie but as a result, the Archie equation method yields the best-fit result in the given reservoir formation of the Area. We can also estimate effective saturation concerning total effective porosity [38].

## 4 Results and discussions

### 4.1 Seismic section interpretation

The available seismic data consist of time-migrated seismic sections (i.e., PSTM version) whereas available seismic sections are useful fold data with low to medium signal-noise ratio.

**Fig. 8:**
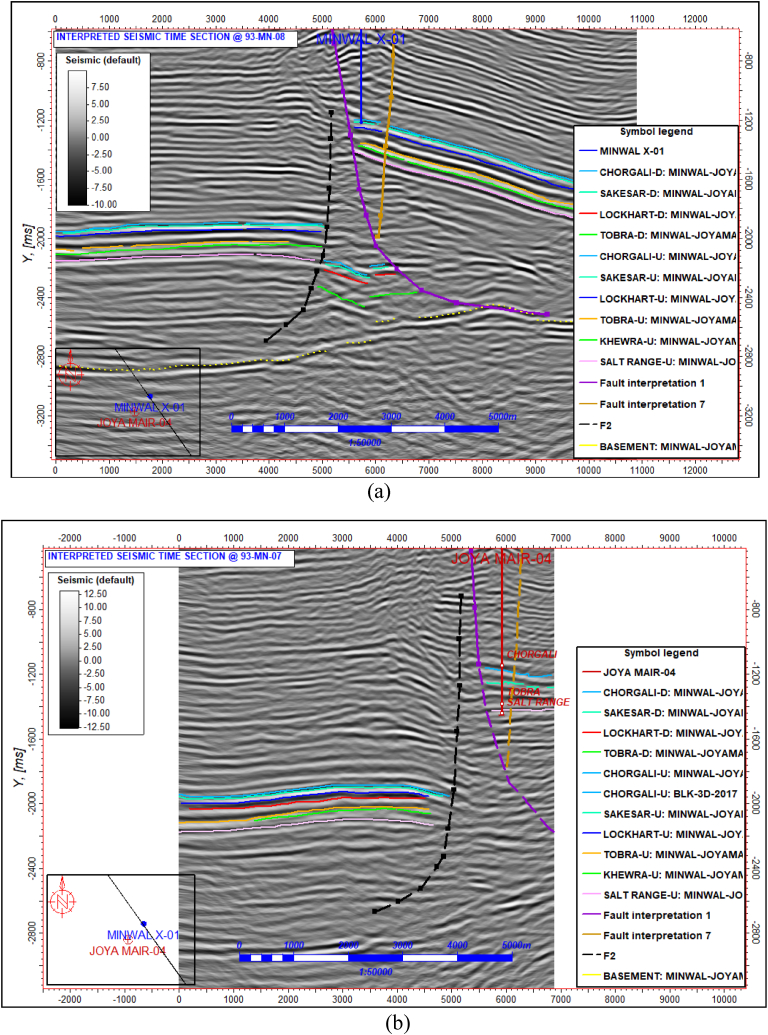
(a) Interpretation of time section for line 93-MN-08-PRESTM. (b) Interpretation of time section for line 93-MN-07-PRESTM.

All prospective horizons were marked on the seismic section which includes Chorgali, Sakesar, Patala, Lockhart, Tobra, Khewra, and Salt Range. These all-marked horizons were mapped for fault correlation.93-MN-08 is the control line through Minwal X-01 well. Interpretation started from this line and all horizons were marked along with major and sub-thrust faults (Fig. 8a). The line 93-MN-07 is also the control line passing through the Joyamair-04 well (Fig. 8b). Overall, two major faults were identified, and a triangle zone defined which is the result of thin-skinned deformation [24]. This tringle zone is the principal reason behind the accumulation of oil and gas in this field. The above-mentioned two lines are identical concerning horizon and fault dip/trend. For this reason, it was very helpful to correlate faults and also Mistie's calculations along horizons.

### 4.2 Time contour mapping

All the prospective horizons were interpreted using seismic data and fault correlation maps, including two major thrust faults and some minor back thrusts.

**Fig. 9:**
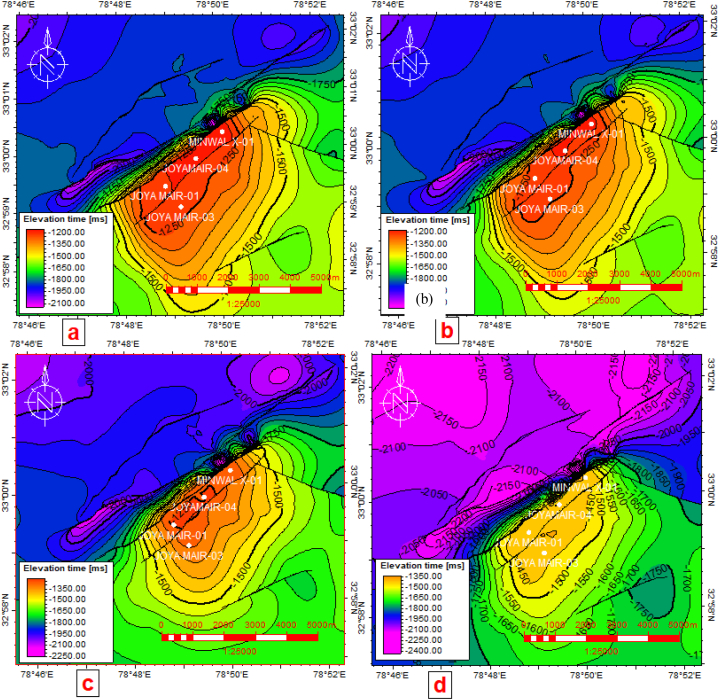
(a) Chorgali Formation TWT structural map. (b) Sakesar Limestone TWT structural map. (d) Lockhart Formation TWT structural map. (d) Tobra Formation TWT structural map.

The time contour maps for Chorgali, Sakesar, Lockhart and Tobra formations were yielded to identify the size and geometry structural closure which is shown in Fig. 9(a–d). Time structural maps clearly indicate the presence of fault-bounded anticline and lower values of fault throw in addition to the thin-skinned deformation in the study area. Overall structure modeling and formations (horizons) mapping indicate that Minwal-Joyamair is a fault-bounded structure that encompasses both fields by one major thrust of NE-SW direction.

### 4.3 Velocity modeling

**Fig. 10:**
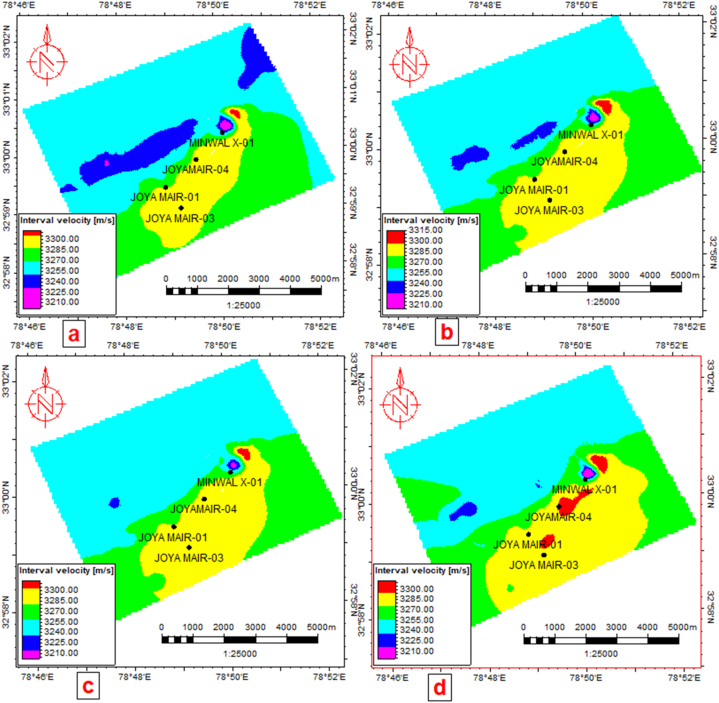
(a) Chorgali interval velocity map. (b) Sakesar Limestone interval velocity map. (c) Lockhart interval velocity map. (d) Tobra interval velocity map.

A velocity model has been generated using seismic-to-well Time Depth Relation (TDR), which was further used for geophysical data conversion. Seismic velocity analysis shows that velocity variation is from 3200 m/s to 3300 m/s as Eocene formations have a lower velocity range than the deeper formations of the Cambrian formation. The interval velocity mapping of Chorgali, Sakesar, Lockhart and Tobra formations is shown in Fig. 10(a–d).

As used a linear regression method for velocity estimation, variation is linear and depth. The limitation was present due to the availability of seismic time-depth relation which was only present in two wells (i.e., Minwal X-01 and Joyamair-04). Meanwhile, for deeper horizons of the Cambrian age, only Joyamair-04 well data was available for velocity estimation.

### 4.4 Depth contour maps

The accuracy and consistency of the information interpreted from seismic data in the time domain and well data in the depth domain contribute to reservoir characterization. These are obtained by the integration of seismic and well-logging data. It is a key point to obtaining accurate conversion. Well-log data is mostly very sparse and to a limited area in whole field depth conversion. Thus, well-log data is mostly used to depict functions related to interval velocity according to the study area's regional geology.

Minwal and Joyamair consist of one main structural petroleum system, which consists of fault-bounded low to mid-angle thrust faults. There are two main thrust faults which are the Northeast-Southwest strike and dipping toward the southeast, this is the main structural fault that encompasses the structural high of this field.

The main primary reservoir of the field is Chorgali, Sakesar, and Lockhart formations; all of these formations were interpreted and mapped using seismic and well-log data. Instead of the primary reservoir of the Eocene and Paleocene, Tobra Formation can also be productive for hydrocarbon exploration.

**Fig. 11:**
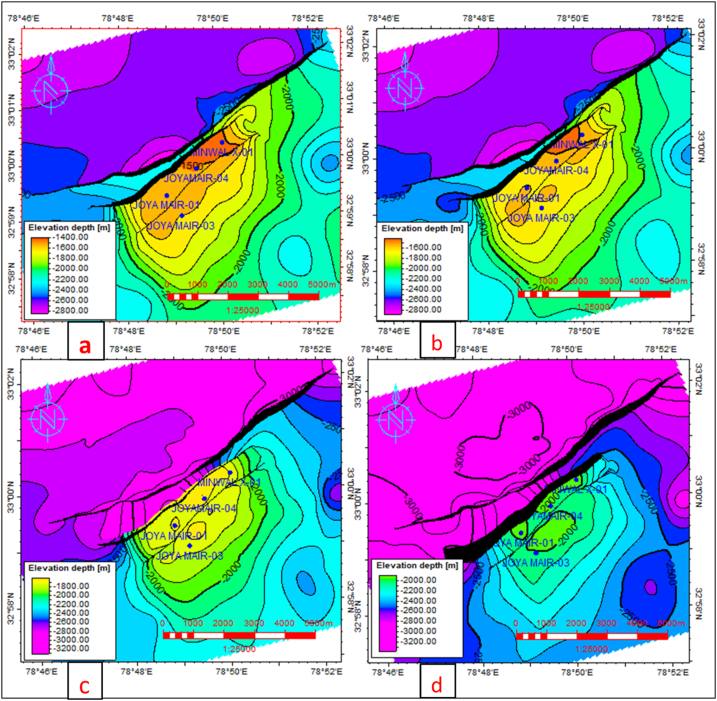
(a) Chorgali (reservoir) Formation depth contour map (b) Sakesar (reservoir) Limestone depth structure map. (c) Lockhart (reservoir) Formation depth structure map. (d) Tobra (reservoir) Formation depth structure map.

Chorgali and Sakesar formations of the Eocene age are the shallowest reservoirs with an average true vertical depth (TVD) of 1500–1600 m at structural high (Fig. 11a). One back thrust dipping toward the northeast is also marked which is tied to the main thrust fault of the southeast dip (Fig. 11b). Lockhart Formation (reservoir) depth structure map is shown in Fig. 11c. The depth of Tobra (potential reservoir) Formation in the Minwal field is less than 1800 m SSTVD reservoir can be potential for future field development as like the surrounding fields (Fig. 11d). The downthrown part of both fields is about 3000 m, which can be potential for deeper prospects. The thrown of major thrust is very high so hydrocarbon migration via major fault cannot be possible. However, risk can be involved when going for deeper potential due to high temperature and pressure constraints can be possible.

## 5 Petrophysics

Exploring the carbonate reservoir system is very important in an era of higher demand for resources and progressively advances in containing reserves. Carbonate reservoir systems are more complex than clastic reservoirs because of a complication occurring at all measurement scales. Complexity in carbonates can be associated with facies variation, lithological heterogeneity, mineral composition, sediment connectivity, and textures. Each of these can be related to different depositional environments and digenesis processes. Much research has been carried out to understand these problems, which evaluate these carbonate reservoirs [[39], [40], [41], [42], [43], [44]].

### 5.1 Identification of lithology and M − N plot

**Fig. 12:**
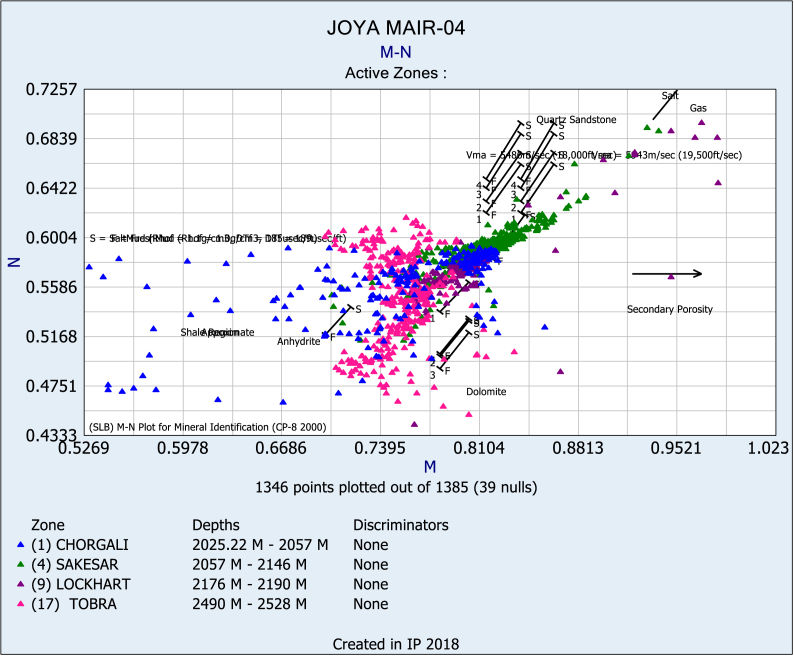
M − N plot for the Chorgali, Sakesar, Lockhart and Tobra formations.

Identifying lithology from well logs in carbonate reservoirs can be a challenging task due to the complex mineralogy, heterogeneity, and variable porosity and permeability of these reservoirs. However, there are several well logs that can provide useful information for lithology identification in carbonate reservoirs. Identification and discrimination of lithology are very crucial, especially in a carbonate reservoir as carbonate reservoir tend to have very complex facies and diagenetic features which can ultimately makes them a very good or a poor reservoir. For this purpose, it was considered not only lithology identification by conventional methods but also use other methods that may help in identifying prospective zones [[45], [46], [47], [48]]. In this study different well logs are used for lithological prediction as M − N plots for the Chorgali, Sakesar, Lockhart, and Tobra formations (Fig. 12).

M − N cross plot have been generated using density (RHOB), neutron (NPHI) and sonic (DT) logs to identify binary and ternary mixtures of minerals. The main objective of M − N cross plot is to identify the zones of different lithology on the basis of difference in mineral composition [39,40]. These values are calculated by using equations (5), (6).

(5) $$M=\frac{\Delta tf-\Delta tma}{\rho b-\rho f}x\;0.01$$

(6) $$N=\frac{\emptyset NF-\emptyset Nma}{\rho b-\rho f}$$

Where Δtf = transit time of fluid (189 for fresh mud), Δtma = transit time of the matrix, ρb = bulk density of formation, ρf = density of fluid (1.0 for fresh mud), ∅NF = neutron porosity of fluid (use 1.0), and ∅Nma = neutron porosity of the matrix.

The cross plot identifies the different zones of reservoir having different values for M − N where clastics of Tobra Formation having low values whereas carbonates of Chorgali formation having highest values.

### 5.2 Volume of shale

**Fig. 13:**
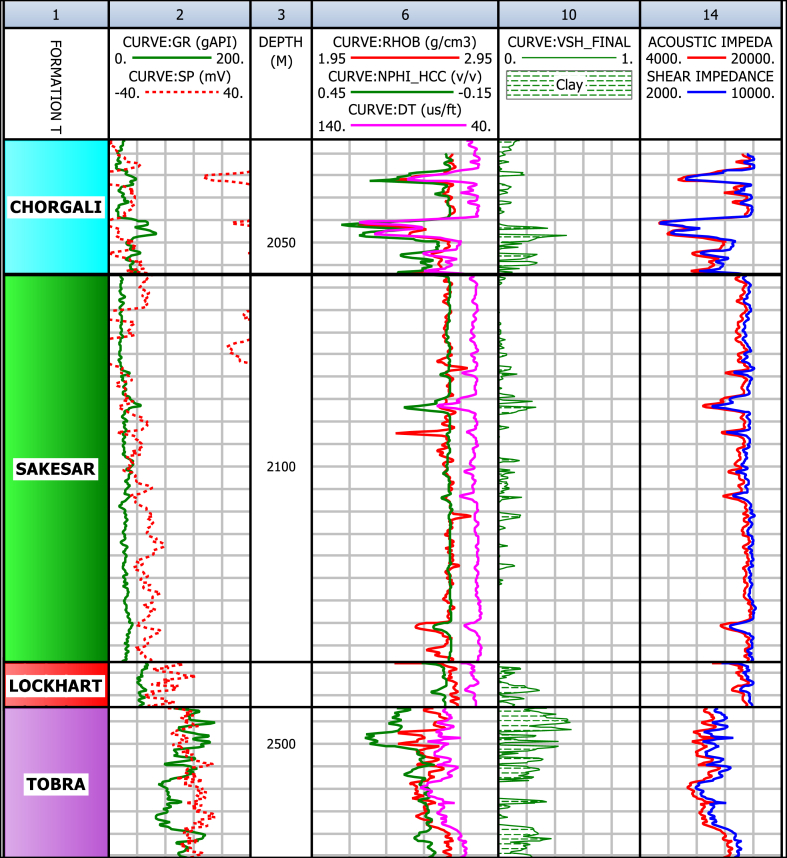
Identification of shale and low porosity zones using rock physics properties of Joyamair-4 well.

The shale volume means the total volume occupied by the clay-rich minerals in a reservoir. Existence of shale (clay-rich rock) in a formation can greatly affect the different fundamental petrophysical parameters of a reservoir such as its total porosity, effective porosity and permeability etc. [39,40]. The shale volume was calculated using the volume of shale index by linear equation methods, which results in the identification concentration of shale content, as shown in Fig. 13. Calculation of the volume of clay/shale shows that the lower part of Chorgali and the upper part of Sakesar Limestone have high values of shale, which is a seal or barrier for hydrocarbon. The shale in Chorgali (3%) and Sakesar (4%), which is low, is also considered a source of hydrocarbon generation (Table 1).

**Table 1:** Petrophysical results for Joyamair-04 well.

Formation	Depth (m)	Volume of shale (%)	Effective Porosity (%)	Total Porosity (%)	Saturation of Water (%)	Saturation of hydrocarbons (%)
Chorgali	2025–2057	3.3	5.94	7.27	55	45
Sakesar	2057–2146	3.78	1.28	1.68	53.89	46.11
Lockhart	2176–2190	9.79	2.87	4.73	74.8	25.2
Tobra	2490–2528	28	5.70	9.52	26	74

In the case of known carbonate reservoirs, there is often a good relationship between acoustic impedance and porosity. The best seismic attributes to be correlated to average porosity values require a detailed evaluation, but usually, the Acoustic Impedance (AI) over the reservoir interval provides good results [49]. Moreover, petrophysical and rock physics parameters can also be helpful not only to identify the zones of shale but also the mark the zones of low porosity. For this reason, a relation between the volume of shale and calculated acoustic impedance was used to mark the zones of high shale content with low porosity (Fig. 13).

### 5.3 The porosity of the reservoirs

**Fig. 14:**
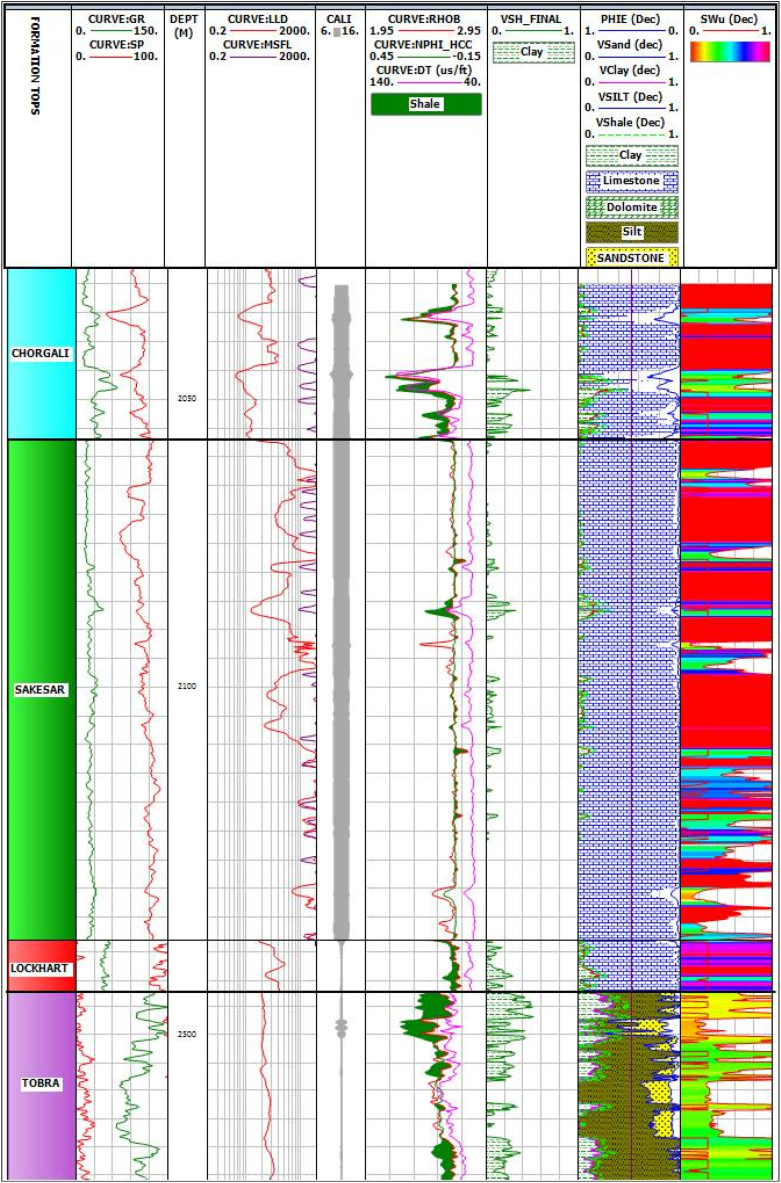
Saturation of water calculation for the Chorgali, Sakesar, Lockhart and Tobra formations for Joyamair-04 well.

Identifying lithology is crucial for reservoir characterization as it affects petrophysical parameters such as porosity, permeability, and fluid saturations. The lithology of the Chorgali, Sakesar, Lockhart, and Tobra formations was determined using gamma ray logs, and cross-plots were utilized to define lithology and petrophysical parameters. Neutron-density cross-plot was employed to identify lithology and porosity of the formations [50]. Neutron-density cross-plots were employed to identify lithology and porosity of the formations. Available pore space for the storage within Chorgali, Sakesar, Lockhart, and Tobra formations were determined from the available log suite which shows the estimated values of total porosity as 7%, 2%, 5%, and 10%, respectively (Table 1; Fig. 14). The respective values of effective porosity for these rock formations are 6%, 1%, 3% and 6%. For the calculation of porosity one single parameter is not sufficient for the best result whereas a cross plot of different petrophysical properties can be very helpful. For the same reason, density and porosity cross-plots can be very helpful in lithological identification.

### 5.4 Saturation

Water saturation is a vital component in the general description and further classification of any reservoir. Multiple methods/equations have been adopted which have been utilized to determine water saturation, but water saturation values show a drastic change to the petrophysical properties and also the facies variation in reservoir formations. Due to this fact, various methods have been used to represent the reservoir and predict how the reservoir will behave under certain conditions.

Resistivity logs have been consistently used to determine the saturation of water in the reservoirs by making use of Archie's equation [51] which shows a relationship between water saturation to the true permeable formation resistivity, the formation porosity, and the formation of water resistivity. Saturation of water has been calculated using Archie's equation for all the reservoirs of Eocene (Chorgali, Sakesar), Paleocene (Lockhart), and Permian (Tobra) as shown in Fig. 14. The hydrocarbon saturation values for these formations are 45%, 46%, 25% and 74%, respectively. Tobra formation shows the maximum value of hydrocarbon saturation as well as the effective and total porosities which highlights its potential to be a good reservoir. All the petrophysical results are combined in a single table as shown in Table 1.

## 6 Conclusions

• Hydrocarbon potential of developed Field of Minwal-Joyamair for any future prospectivity has been done using all the available seismic and well data. The limitation was present by the availability and quality of seismic and well data.
• In contrast, all the available data were incorporated for a better understanding of the reservoir potential of Cambrian to Eocene reservoirs through geological and geophysical data. Distribution of every petrophysical and seismic-related research has been done, giving a better idea of field behavior and fluid distribution.
• The primary reservoir of the study area is fractured carbonate (i.e., Chorgali, Sakesar, and Lockhart) formations with secondary clastic reservoirs of Tobra which still need to be explored and produced. The quality of sand is good in Tobra formation. Cementing material in Tobra is calcareous, which helps improve production through stimulation. Based on qualitative interpretation, the reservoir permeability in Tobra Formation is good.
• Reservoir correlation with adjoining producing fields indicates good reservoir potential at Tobra levels. The petrophysical analysis also shows favorable hydrocarbon saturation in Tobra reservoirs.

## Funding

Open access funding is provided by University of Lausanne.

## Data availability statement

The authors do not have permission to share data.

## Author contribution statement

Muhammad Ali Umair Latif, Muhsan Ehsan, and Abid Ali: Conceived and designed the experiments; Performed the experiments; Analyzed and interpreted the data; Contributed reagents, materials, analysis tools or data; Wrote the paper. Muhammad Ali, Armel Zacharie Ekoa Bessa and Mohamed Abioui: Contributed reagents, materials, analysis tools or data; Wrote the paper.

## Declaration of competing interest

The authors declare that they have no known competing financial interests or personal relationships that could have appeared to influence the work reported in this paper.
